# Mechanical-force-induced non-local collective ferroelastic switching in epitaxial lead-titanate thin films

**DOI:** 10.1038/s41467-019-11825-2

**Published:** 2019-09-02

**Authors:** Xiaoyan Lu, Zuhuang Chen, Ye Cao, Yunlong Tang, Ruijuan Xu, Sahar Saremi, Zhan Zhang, Lu You, Yongqi Dong, Sujit Das, Hangbo Zhang, Limei Zheng, Huaping Wu, Weiming Lv, Guoqiang Xie, Xingjun Liu, Jiangyu Li, Lang Chen, Long-Qing Chen, Wenwu Cao, Lane W. Martin

**Affiliations:** 1 School of Civil Engineering, Harbin Institute of Technology, Harbin, 150001 China; 2 School of Materials Science and Engineering, Harbin Institute of Technology, Shenzhen, 518055 China; 3 Departments of Materials Science and Engineering, University of Texas at Arlington, Arlington, TX 76019 USA; 4 Department of Materials Science and Engineering, University of California, Berkeley, CA 94720 USA; 5 X-ray Science Division, Advanced Photon Source, Argonne National Laboratory, Argonne, IL 60439 USA; 6 School of Materials Science and Engineering, Nanyang Technological University, Singapore, 639798 Singapore; 7 Condensed Matter Science and Technology Institute, Harbin Institute of Technology, Harbin, 150080 China; 8 Key Laboratory of E&M (Zhejiang University of Technology), Ministry of Education & Zhejiang Province, Hangzhou, 310014 China; 9 Department of Mechanical Engineering, University of Washington, Seattle, WA 98195-2600 USA; 10 Department of Physics, Southern University of Science and Technology, Shenzhen, 518055 China; 11 Department of Materials Science and Engineering, The Pennsylvania State University, University Park, PA 16802 USA; 12 Department of Mathematics and Materials Research Institute, The Pennsylvania State University, University Park, PA 16802 USA; 13 Materials Sciences Division, Lawrence Berkeley National Laboratory, Berkeley, CA 94720 USA

**Keywords:** Ferroelectrics and multiferroics, Ferroelectrics and multiferroics

## Abstract

Ferroelastic switching in ferroelectric/multiferroic oxides plays a crucial role in determining their dielectric, piezoelectric, and magnetoelectric properties. In thin films of these materials, however, substrate clamping is generally thought to limit the electric-field- or mechanical-force-driven responses to the local scale. Here, we report mechanical-force-induced large-area, non-local, collective ferroelastic domain switching in PbTiO_3_ epitaxial thin films by tuning the misfit-strain to be near a phase boundary wherein *c/a* and *a*_1_/*a*_2_ nanodomains coexist. Phenomenological models suggest that the collective, *c*-*a*-*c*-*a* ferroelastic switching arises from the small potential barrier between the degenerate domain structures, and the large anisotropy of *a* and *c* domains, which collectively generates much larger response and large-area domain propagation. Large-area, non-local response under small stimuli, unlike traditional local response to external field, provides an opportunity of unique response to local stimuli, which has potential for use in high-sensitivity pressure sensors and switches.

## Abstract (web summary)

Clamping effects in ferroelestastic thin films limits their usefulness for applications such as sensitive mechanical sensors. Here, the authors report on non-local mechanical force induced switching in PbTiO_3_ thin films by tuning the material to a state of nearly energetically degenerate co-existing domains.

## Introduction

Domain structure and its switching behavior are crucial to material properties, including dielectric and piezoelectric response in ferroelectrics and magnetoelectric coupling in multiferroics^1–3^. In particular, for ferroelectrics, ferroelastic switching (i.e., non-180° switching events) can give rise to large dielectric and electromechanical responses due to strong lattice strain-polarization coupling^4–7^. Furthermore, large-area ferroelastic switching under small stimuli can also be vital for magnetoelectric coupling in multiferroics, which are being considered for low-power electric field-controlled spintronics^8–10^. Large-area ferroelastic switching in ferroelectrics, however, has typically only been observed in bulk materials^11–13^. In fact, it is generally thought that ferroelastic switching is quenched in ferroelectric epitaxial thin films due to substrate constraints^14–16^. In order to reduce substrate clamping and facilitate ferroelastic domain switching, several approaches have been explored^17–24^. For example, by fabricating thin films into micro- or nanoscale islands with lateral dimensions on the order of the thickness of the film, researchers have released lateral constraint from the substrate, thereby enabling larger fractions of ferroelastic switching^4,9^. Such approaches require lithography and/or milling/etching, both of which are time-consuming and challenging. Furthermore, the lateral sizes of the features must be very small, limiting the ability to produce ferroelastic switching across large areas. Therefore, despite considerable efforts, it remains a challenge to achieve large-area ferroelastic switching in ferroelectric epitaxial thin films.

It is also known that long-range interactions (i.e., dipole–dipole electrostatic and elastic interactions) in ferroelectrics could induce collective behavior during domain switching^25–30^. For instance, phenomenological approaches have revealed that the electrostatic interaction between switched nuclei in ferroelectric thin films with 180° stripe domains can be long range and induce collective nucleation/switching, thereby effectively reducing the switching barrier^27^. Moreover, the domain-switching process in ferroelectric ceramics is thought to be a highly correlated collective process such that the switching process in one grain affects that in the neighboring grains because of inter-grain elastic interactions^28^. Such collective behavior and any resulting large responses would be more evident in ferroelectrics perched near a phase boundary wherein two phases are nearly energetically degenerate and can be interconverted by small external stimuli^28–31^. While most observations of collective switching behavior have been observed in bulk or polycrystalline thin films of ferroelectrics^28–30,32^, work in epitaxial thin films has shown that ferroelastic switching, with enhanced piezoelectric response, can be obtained in thin films of tetragonal ferroelectrics such as PbZr_0.2_Ti_0.8_O_3_ by fabrication of micro- or nanoscale islands^3,33^. More recently, ferroelastic switching was also observed in tetragonal ferroelectric thin films when they were grown on the right lattice-(mis)matched substrates^18,19^. At the same time, the use of scanning-probe excitation has also opened the door to induce and control ferroelastic switching via a combination of applied voltages and tip motion^4,34,35^. Despite these advances, however, previous studies have illustrated only local ferroelastic domain-switching behaviors (i.e., directly under or very close to the poling region). It is generally well accepted that such effects will be highly localized as the elastic clamping of the substrate limits the ability to create large-scale ferroelastic changes in the domain structure.

Recent studies have identified epitaxial strain approaches to create multiple nanoscale-domain structures co-existing in PbTiO_3_ thin films^36^ which provides an intriguing system to explore in this regard. More specifically, 40-nm-thick, (001)-oriented films of PbTiO_3_ grown on DyScO_3_ (110)_*O*_ (where the *O* denotes orthorhombic indices) experience a compressive strain that drives the formation of traditional *c*/*a* domain structures, while films grown on NdScO_3_ (110)_*O*_ substrates with large tensile strain exhibit *a*_1_/*a*_2_ domain structures, and films grown on SmScO_3_ (110)_*O*_ substrates with a strain state between that of DyScO_3_ and NdScO_3_ exhibit a coexistence of both *c*/*a* and *a*_1_/*a*_2_ domain variants^36^. In effect, epitaxial strain can be used to place this material on the brink of a transition between domain-structure variants and is an ideal route by which to explore the potential for large responses and collective effects^37,38^.

Here, we focus on PbTiO_3_ epitaxial heterostructures with co-existing *c*/*a* and *a*_1_/*a*_2_ nanodomains by tuning the misfit strain to be near a phase boundary. Electric field-poling studies via scanning-probe microscopy reveal that reversible and localized ferroelastic switching can be achieved by fine control of the out-of-plane poling voltage. Local mechanical force induced by the tip of an atomic force microscope, on the other hand, can drive large-area, non-local ferroelastic switching—much larger than the contact area. Using Landau phenomenological theory including phase-field simulation^39^ and polydomain theory^40^, further insights into the origin of the large-area, non-local, collective ferroelastic switching behavior with respect to co-existing energetically degenerate nanodomain variants are provided.

## Results

### Epitaxial growth of PbTiO_3_ thin films

**Fig. 1:**
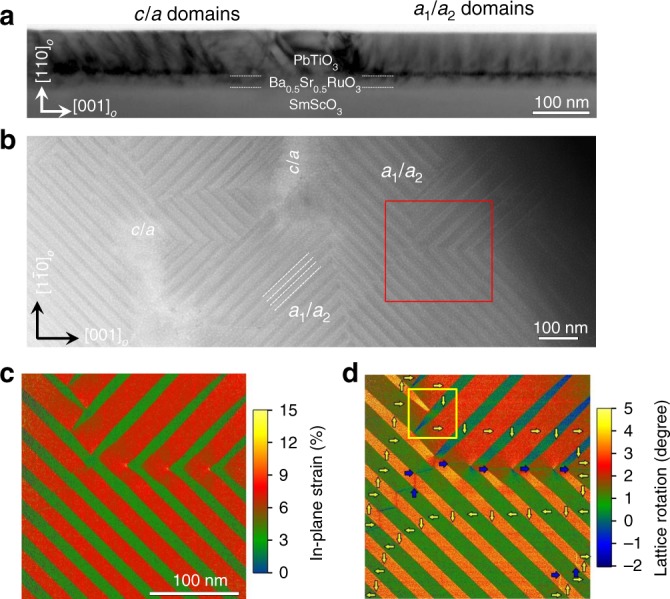
Domain structures of 70-nm-thick PbTiO_3_ films grown on Ba_0.5_Sr_0.5_RuO_3_/SmScO_3_ (110)_*O*_. **a** Cross-sectional transmission electron microscopy (TEM) image of the heterostructures where the white dashed lines mark the PbTiO_3_/Ba_0.5_Sr_0.5_RuO_3_ and Ba_0.5_Sr_0.5_RuO_3_/SmScO_3_ interfaces. **b** Plan-view high-angle annular dark field-scanning transmission electron microscopy (HAADF-STEM) image of the heterostructure where the dashed lines indicate the *a*_1_/*a*_2_ domain walls. **c** In-plane strain map (*ε*_xx_) and **d** lattice rotation map (*ω*) extracted from the HAADF-STEM lattice image via geometrical phase analysis (GPA) for the area in the red square in (**b**). The yellow arrows indicate the head-to-tail structures and the blue arrows indicate 180° domain walls. The lattice rotation at 180° domain walls can be used to identify the polarization directions beside the domain walls. Yellow-border square in (**d**) reveals relatively large fluctuations especially near the needle-shaped domains

70 nm PbTiO_3_/20 nm Ba_0.5_Sr_0.5_RuO_3_/SmScO_3_ (110)_*O*_ heterostructures were deposited by pulsed-laser deposition (“Methods”)^36^. The nominal misfit strain between the PbTiO_3_ film and substrate, controlled to be near a critical tensile strain (Supplementary Fig. 1), is close to the middle of the critical misfit strains of 0.2% (below 0.2%, *c*/*a*-domain structures are favored) and 0.8% (above 0.8%, *a*_1_/*a*_2_-domain structures are favored) where a nearly equal coexistence of the *c*/*a-* and *a*_1_/*a*_2_-domain variants is expected^36^. X-ray diffraction studies and reciprocal space mapping analysis (Supplementary Figs. 2 and 3) reveal the presence of high-quality, epitaxial growth of the single-phase PbTiO_3_ films with *a* and *c* domains. Cross-section, bright-field transmission electron microscopy (TEM) (Fig. 1a) and plan-view high-angle annular dark-field-scanning transmission electron microscopy (HAADF-STEM) (Fig. 1b) studies confirm the presence of *c*/*a*-domain variants with domain walls parallel to the [111]_*O*_ (i.e., [101] in pseudocubic indices), and *a*_1_/*a*_2_-domain variants with domain walls along the $[1\bar 11]_O$ and $\left[ {1\bar 1\bar 1} \right]_O$ (i.e., [110] and $[1\bar 10]$ in pseudocubic indices, respectively) and domain widths of ~30 nm (Fig. 1b). Note that the *a*_1_/*a*_2_ domains are obscured in the cross-section TEM since the *a*_1_/*a*_2_ domain walls are not aligned along the projected zone axis^41^. Likewise, the *c*/*a* domains are obscured in the plan-view imaging as their domain walls run through the thickness of the sample. Local strain fields are assessed by geometric phase analysis (GPA, Gatan Digital Micrograph) and visualized using the Gatan Digital Micrograph software^42^. As compared with the lattice parameters of the bulk counterpart, the in-plane strain (Fig. 1c) and lattice rotation (Fig. 1d) maps extracted from the HAADF-STEM lattice image of the selected area (red square, Fig. 1b) show near perfect periodic elastic fields within the *a*_1_/*a*_2_ domains (head-to-tail domain structures, Fig. 1d). Furthermore, the lattice rotation map reveals relatively large fluctuations especially near the needle-shaped domains (yellow square, Fig. 1d), suggesting a high degree of structural softness (Fig. 1d)^31^. Previous studies in ferroelectric KH_2_PO_4_ crystals found that the interaction between such needle-shaped domain tips is long range^43,44^. The observed needle domains in these PbTiO_3_ films may also enhance the mobility of domain walls and long-range interactions^42,45^. The co-existing domain structure is further confirmed by piezoresponse force microscopy (PFM) studies (Supplementary Fig. 4). The well-ordered *c*/*a* and *a*_1_/*a*_2_ nanodomains observed in the PbTiO_3_ thin films should naturally have strong elastic interactions between neighboring domains due to the large crystal anisotropy, which, in turn, may facilitate collective switching behavior in the ferroelectric/ferroelastic system.

### Reversible control of ferroelastic switching by out-of-plane electric fields

Earlier reports on (001)-oriented tetragonal ferroelectric thin films, such as PbTiO_3_ and PbTi_0.8_Zr_0.2_O_3_ films grown on SrTiO_3_ (001) substrates^14^, indicate that it is challenging for large-area ferroelastic switching between *a* and *c* domains using out-of-plane electric fields because the compressive strain imposed by the substrate strongly (elastically) favors *c* domains. If the energy barrier between the in- and out-of-plane polarized domains is made small, however, by, for example, tuning the misfit strain, ferroelastic switching could occur^36^. Furthermore, large-area, even non-local, ferroelastic switching could be enabled when the film is tuned close to the domain-structure boundary.

**Fig. 2:**
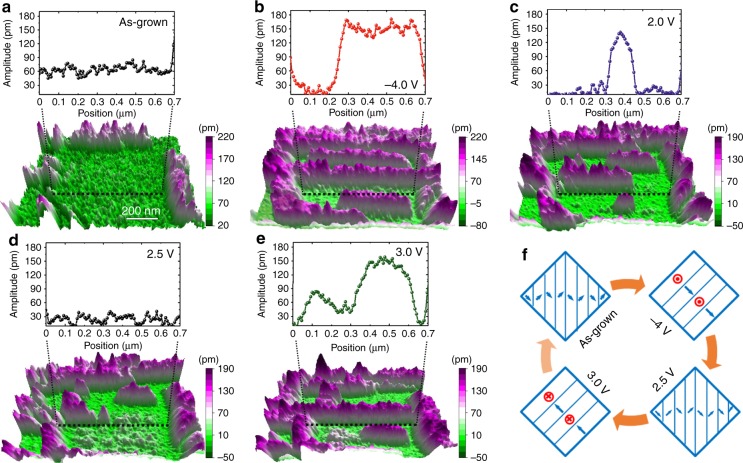
Ferroelastic switching via out-of-plane electric field poling. 3D representation of the out-of-plane piezoresponse force microscopy (PFM) amplitude and corresponding line trace along the noted dashed lines in (**a**), as-grown state, **b** after being poled upward via a −4 V bias, **c** after an applied +2 V bias, **d** after an applied +2.5 V bias, and **e** after further increasing bias to +3 V. **f** Schematic illustration of the domain-structure evolution under stepwise electric field

To explore this idea, we first applied different out-of-plane DC voltages using a PFM tip in a representative 1.2 × 1.2 µm area (Fig. 2a–e). Upon poling the entire area with a −4 V bias applied through the tip, the majority of the *a*_1_/*a*_2_ domains in the as-grown state (with weak out-of-plane PFM response, Fig. 2a) reorganize into *c/a* domains (with strong out-of-plane PFM response and upward pointing *c* domains, Fig. 2b). In order to investigate the reversibility of the ferroelastic switching, stepwise positive bias voltages were applied in an attempt to switch the induced *c/a*-domain structures back to *a*_1_*/a*_2_-domain structures. At a +2 V applied bias, the *c/a*-domain structures begin to switch back into *a*_1_/*a*_2_ domains (with low vertical amplitude, Fig. 2c). By +2.5 V applied bias, the upward pointing *c*-domain regions gradually disappear (Fig. 2d). Upon further increasing the poling voltage to +3 V, the *a*_1_/*a*_2_-domain structures are again interconverted back to *c/a*-domain structures, but this time with the *c* variants poled downward (Fig. 2e). As revealed above, the domain evolution under stepwise voltage suggests that ferroelastic switching readily occurs and is reversible as per the process described herein (Fig. 2f). Although *c/a* domains can be switched back to *a*_1_/*a*_2_ domains by application of large in-plane voltage (which also requires lithographic patterning) as illustrated in earlier studies^36^, it is interesting to note that one can also switch the *c/a* domains back into *a*_1_/*a*_2_ domains step-by-step with a small and simple out-of-plane electric field. This further suggests the rather small energy barrier between these domain structures in the films.

### Mechanical force-induced non-local ferroelastic switching

The above demonstration of reversible *a*_1_/*a*_2_ to *c*/*a* ferroelastic switching by purely out-of-plane electric fields, however, is found to be localized, probably due to weak electrostatic interactions. To trigger a larger area non-local switching, an appropriate driving force, something more influential than electrostatics, is required. In this regard, compared with electric field, mechanical force has the potential to provide the long-range elastic interaction necessary to drive these effects^46^. It is important to note that several studies of mechanical force-induced domain switching in ferroelectric thin films (typically due to flexoelectric effects) have been reported^47,48^. Only local response to the applied mechanical force, however, was observed in those studies. This is again due to the large compressive misfit strain which likely favors *c* domains and thus hampers ferroelastic switching. To enable collective domain switching, the system should be carefully tuned to the brink of a structural instability, as we have achieved here by delicately controlling the epitaxial strain.

**Fig. 3:**
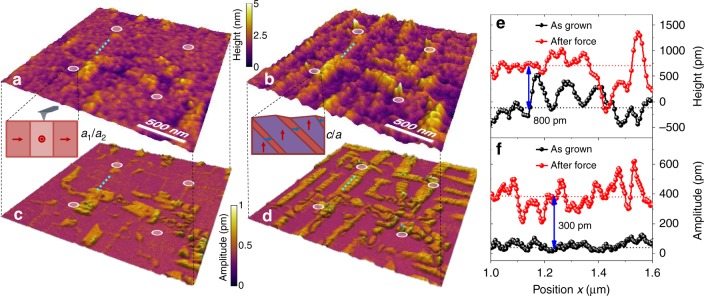
Mechanical force-induced non-local, large-area ferroelastic switching. The force mapping is completed in a 2 × 2 array of points (the tip radius is ~25 nm) at the corners of a 1 × 1 µm area within a 2 × 2 µm scanned area. Topographic images of the films **a** before and **b** after the application of local four-point force as noted by four shaded circles. Out-of-plane piezoresponse force microscopy (PFM) images in **c** as-grown state and **d** after the four-point force mapping. A dramatic change in the domain structure occurs outside the tip-sample contact area and appears in both the topography **b** and out-of-plane PFM amplitude **d** images after the application of ~600 nN force to the noted positions. Corresponding line profile changes in **e** surface height and **f** out-of-plane PFM amplitude as indicated by the dashed lines in (**a**–**d**). The average height and out-of-plane PFM amplitude of the switched area are ~800 pm and ~300 pm higher than those of the as-grown *a*_1_/*a*_2_ domains, respectively

We apply a point array of force using an atomic force microscope Fmap (“Methods”) on an area of the film which possesses a majority of *a*_1_/*a*_2_ domain areas (Supplementary Fig. 4). The force mapping is completed in a 2 × 2 array of points (the tip radius is only ~25 nm) at the corners of a 1 × 1 µm area within a 2 × 2 µm scanned area (Fig. 3a–d). After the application of a setpoint with voltage of 2 V, corresponding to a force of ~600 nN (“Methods”), to the noted positions, a dramatic change in the domain structure occurs even outside the tip-sample contact area and appears in both the topography (Fig. 3a, b) and out-of-plane PFM amplitude (Fig. 3c, d) images. More specifically, it is found that the majority of the initial *a*_1_/*a*_2_-domain structures across the entire scanned area are converted to *c/a*-domain structures. We note that the changes can extend across nearly the entire 2 × 2 µm scanned area (Fig. 3b, d, and Supplementary Fig. 5). These changes are made more evident by extracting the evolution of the surface morphology and out-of-plane PFM amplitude (dashed lines, Fig. 3a–d) across these switched areas (which is outside the tip-sample contact region). After applying the local mechanical force, the average height and out-of-plane PFM amplitude of the switched area are ~800 pm and ~300 pm higher than those of the as-grown *a*_1_/*a*_2_ domains (Fig. 3e, f), respectively; clearly demonstrating that large-area, non-local ferroelastic switching occurs (Supplementary Figs. 6–8).

### Phase-field simulations of the domain switching under tip-induced mechanical force

**Fig. 4:**
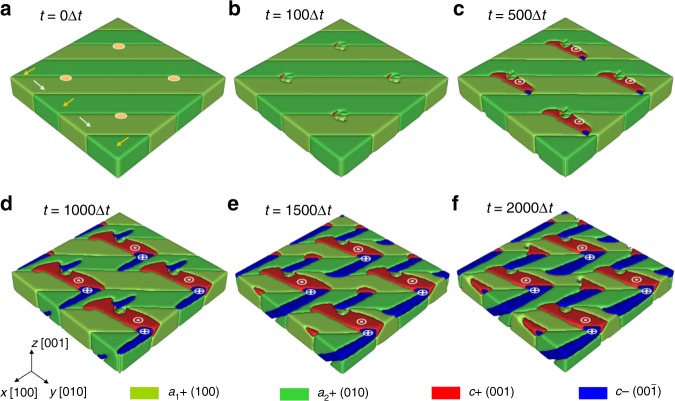
Phase-field simulations of mechanical force-induced domain evolution. The films with *a*_1_/*a*_2_ domains are subjected to 0.5% tensile strain. **a** As-grown *a*_1_/*a*_2_ state at *t* = 0∆*t* with illustration of probe tip force locations as noted by four circles, and arrows indicate the polarization directions of *a*_1_/*a*_2_ domains, **b** *t* = 100∆*t* where *c* domains emerge around the tip-sample contact area, **c** *t* = 500∆*t* where more *c* + domains (red area, polarization up, noted with⊙) are formed and *c* domains (blue area, polarization down, noted with⊕) adjacent *c* + domains begin to emerge, **d**, *t* = 1000∆*t* where *c* domains penetrate all the way down to the bottom, **e**, *t* = 1500∆*t* and **f**, *t* = 2000∆*t* where more *c* domains begin to form, resulting *c/a* domains structures

To understand the large non-local response observed herein, we employed phase-field simulations (“Methods”) to model the domain switching under tip-induced mechanical force^49^. Since the observed non-local response occurs in the areas where *a*_1_/*a*_2_ domains dominate, we start from a quasi-stable state of *a*_1_/*a*_2_ domains under a 0.5%-strain state (time step 0, Fig. 4a). Upon application of the four-point 600 nN tip force in a 2 × 2 array, non-local *a*_1_/*a*_2_ to *c/a* switching gradually occurs which eventually penetrates through the entire film thickness (with *c/a* walls oriented 45° in the cross-section *x-z* plane) (Fig. 4), consistent with the experimental results. After applying the local mechanical force, the initial *a*_1_/*a*_2_ domains beneath the probe tip remain unchanged because the large, tip-induced out-of-plane compression would favor *a* domains with in-plane polarization (Supplementary Fig. 9). The lattice of as-grown, in-plane polarized *a* domains under the tip-induced pressure will slightly expand along the in-plane direction^50^. To match the epitaxial tensile strain, *c* domains are formed adjacent to the *a* domains to locally decrease the average in-plane lattice parameter, resulting in head-to-tail *c/a*-domain structures. Moreover, near the tip-sample contact area, strain-gradient induced, out-of-plane flexoelectric fields would also favor the formation of *c* domains (Supplementary Fig. 10)^51^. Under these combined effects, more *c* domains emerge at the top surface of the film near the tip-sample contact area (Fig. 4b, c). Eventually, *c* domains penetrate all the way across the film over a large area to maintain the lowest total free energy (Fig. 4d–f). Based on the phase-field simulations, the Landau free energy and electrostatic-energy density both decrease during the formation of the *c* domains, while the gradient-energy density increases due to the formation of domain walls (Supplementary Fig. 11). This domain propagation is also evident in the in-plane tensile strain fields, which expand gradually into a large area corresponding to the formation of the *c* domains (Supplementary Fig. 12). Again, to match the epitaxial tensile strain, *a* domains are favored next to *c* domains, then, followed with *a* domains. Since the surrounding *a*_1_/*a*_2_ domains are already relatively unstable under the tensile stress surrounding the probe tip, the system is driven into successive switching with energetically favorable head-to-tail *c/a* configurations co-existing with remaining *a*_1_/*a*_2_ domains until a new energy equilibrium is reached. Since the structure prefers to maintain head-to-tail domain structures to minimize the total energy, the change of domain structures in a local area will release energy and eventually propagate across long distances, leading to collective switching.

## Discussion

Such a collective domain switching process is strongly related to the potential energy barrier between the two domain-structure variants. Based on Landau-type phenomenological theory for polydomain structures^38^, the calculated Landau free-energy densities of the *a*_1_/*a*_2_ and *c/a* domain patterns are equal at the critical strain of ~0.46% at 300 K; however, at high temperature, such as 500 K, are about −22 MJ/m^3^ and −19 MJ/m^3^, respectively, with an energy difference of only ~3 MJ/m^3^ (Supplementary Fig. 13). The transition barrier between the *a*_1_/*a*_2_- and *c/a*-domain patterns is much lower than the energy required for the polarization switch, such as the *c*^+^ to *c*^–^, of about 48 MJ/m^3^ (Supplementary Fig. 13). Such a small energy barrier between the *a*_1_/*a*_2_- and *c/a*-domain variants gives the possibility of domain switching propagation. Since the initial domains formed in the as-grown heterostructures are dominated by the *a*_1_/*a*_2_ domains due to the relatively lower free-energy potential, external stimuli that overcomes the initial energy barrier will force the system to reconfigure into a more favorable state (i.e., co-existing *a*_1_/*a*_2_ and *c/a* domains). Specifically, the mechanical force applied by the probe tip can gently lower the energy barrier and drive the system to a final state favoring a domain pattern with the coexistence of *a*_1_/*a*_2_ and *c/a* domains.

**Fig. 5:**
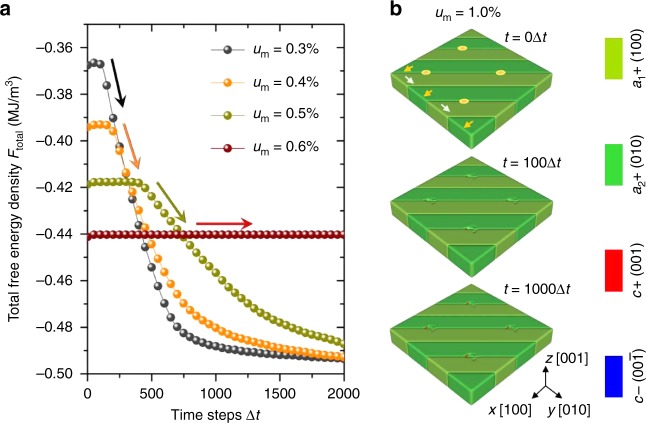
Phase-field simulation of domain evolution under various strain states. **a** Total free-energy density changes after the probe tip-induced force exerted on an initially pure *a*_1_/*a*_2_ domain structure of the films subjected to various tensile strains ranging from + 0.3% to 0.6%. The corresponding arrows indicate the steepness of the energy changes. **b** Phase-field simulations of mechanical force-induced domain evolution of the films with *a*_1_/*a*_2_ domains subjected to 1% tensile strain. Probe tip force locations are noted by four circles on the film surface

This is further supported by phase-field simulations of similar films under different strain states. When the misfit tensile strain is <0.2%, the *c/a* domains dominate and the *a*_1_/*a*_2_-domain structure is unstable even before applied tip forces (Supplementary Figs. 14a–c). For films under misfit strains > 0.3%, however, the pure *a*_1_/*a*_2_-domain structures are in quasi-steady state (Supplementary Fig. 14d–e). We also note that the thickness effect in the phase-field simulation is small (Supplementary Fig. 15). In a small range of misfit strains (0.3–0.5%), non-local response is triggered upon the application of tip pressures, accompanied with a sudden decrease of the total free energy, indicating that the newly formed co-existing *c/a* and *a*_1_/*a*_2_ domains have lower total free energy than the original *a*_1_/*a*_2_-domain structures (Fig. 5a). It is found that the *a*_1_/*a*_2_ domains become more favorable with increasing tensile strain, and thus the initial total free energy density (*t* = 0) decreases with increasing misfit strain (*u*_m_), and the decrease of the total average free-energy density becomes smaller with subsequent collective switching. Such collective switching disappears upon further increasing the misfit strain. For example, films under a tensile strain of 1% have an equilibrium state of *a*_1_/*a*_2_ domains and only subtle changes exist near the tip contact area (Fig. 5b). Ultimately, both the experiments and phase-field simulations reveal that the delicately balanced elastic field in such systems with co-existing domains is crucial for the large response since the elastic field in the film can be easily rebuilt and extended under external perturbation.

In summary, we have successfully demonstrated a long-range, non-local ferroelastic switching behavior in tensile-strained PbTiO_3_ thin films on SmScO_3_ (110) substrates wherein *a*_1_/*a*_2_- and *c/a*-domain structures coexist. Due to the low-energy barrier between the energetically degenerate periodic domain structures, an exotic mechanical force-induced ferroelastic switching with an area much larger than the direct contacting points is observed, accompanied by a large change in topography and piezoelectric response. Phase-field simulations reveal that the nearly energetically degenerate nature of the different domain states is the cause of the delicately balanced elastic field, which is responsible for the large response since the elastic field can be easily rebuilt and extended under external perturbations. Our results pave a new way for possible applications in sensitive mechanical sensors and switches by manipulating the ferroelastic switching behavior of ferroelectric thin films.

## Methods

### Thin film synthesis

Heterostructures were grown via pulsed-laser deposition using a KrF excimer laser (248 nm, Compex, Coherent). Epitaxial 70-nm-thick PbTiO_3_ films were grown on 20 nm Ba_0.5_Sr_0.5_RuO_3_/SmScO_3_ (110)_*O*_ substrates (CrysTec GmbH, Germany) from ceramic targets. The Ba_0.5_Sr_0.5_RuO_3_ layer was grown at a temperature of 750 °C in a dynamic oxygen pressure of 20 mTorr at a laser repetition rate of 3 Hz and a laser fluence of 1.9 J/cm^2^. The PbTiO_3_ layer was grown at a temperature of 675 °C, in a dynamic oxygen pressure of 50 mTorr at a laser repetition rate of 10 Hz, and a laser fluence of 1.9 J/cm^2^. Following growth, the heterostructures were cooled down to room temperature at a rate of 20 °C/min, in a static oxygen pressure of 700 Torr.

### Structural characterization via X-ray diffraction

$\theta - 2\theta$ measurements were carried out using a Panalytical X’pert Pro diffractometer with Cu $K_{\alpha _1}$ radiation (wavelength *λ* of 1.54056 Å). X-ray reciprocal space mapping studies were carried out at the Sector 33-BM-C beamline of the Advanced Photon Source, Argonne National Laboratory.

### Scanning transmission electron microscopy (STEM)

Cross-sectional and plan-view samples were prepared by slicing, grinding, dimpling, and finally ion-milling using a Gatan PIPS, while the plan-view samples were milled only from the substrate side. The HAADF-STEM images were obtained by using a Titan G2 60e300 microscope with a high-brightness field emission gun and double aberration (Cs) correctors from CEOS operating at 300 kV. Strain fields were deduced by using custom plugins of GPA for the software Gatan Digital Micrograph. The visualization of strains and lattice rotations were carried out using the Gatan Digital Micrograph software.

### PFM studies and force mapping

The PFM studies were carried out on a MFP-3D, (Asylum Research) in DART mode using Ir/Pt-coated conductive tips (Nanosensor, PPP-NCLPt with spring constant $k_{{\mathrm{tip}}}$ of ~3 Nm^−1^ and AmpInvOLS of 100.55 nm/V). The tip spring constant $k_{{\mathrm{tip}}}$was calibrated by using a quartz plate based on the formula $k_{{\mathrm{tip}}} = k_{{\mathrm{std}}}\left( {\frac{{InvOLS_{{\mathrm{std}}}}}{{InvOLS_{{\mathrm{tip}}}}} - 1} \right)$ with$InvOLS_{{\mathrm{tip}}}$ the inverse optical lever sensitivity (with units of nm/V) for the cantilever under test measured on a very stiff surface, and $InvOLS_{{\mathrm{std}}}$ the same quantity measured on a compliant surface with spring constant $k_{{\mathrm{std}}}$. We note that the software Igor Pro 6.37 (Asylum Research) can determine the cantilever’s spring constant based on the equi-partition theorem following a 3-step procedure in the Manual of spring constant determination (MFP-3D™ Procedural Operation ‘Manualette’ Version 10 (v080501; Igor 6.04 A)). The applied driving voltage (relative trigger point) of 2 V corresponds to the force of 600 nN with the tip spring constant of about 3 Nm^−1^ (Nanosensor PPP-EFM) based on the formula $F = k_{{\mathrm{tip}}} \cdot AmpInvOLS \cdot V$. We note that the force mapping uses direct current (DC) voltage, while the PFM imaging scan uses alternating current (AC) driving voltage of 1 V in dual AC resonance tracking (DART) mode with smaller contact force.

Force mapping was completed with a 2 × 2 array of points on the corners of the mapping area. First, the tip was lifted to a distance of 1.5 µm above the film surface, then fast driven down to the point with a velocity of 2.98 µm/s. After a dwell time set as 0.99 s, the tip was then lifted and moved to the second point in the order of from top-to-bottom and from left-to-right in each row with velocity of 1.5 µm/s.

### Phase-field simulations

In the phase-field simulations of the domain switching under mechanical tip pressure, we use polarization vector ${\mathbf{P}} = \left( {P_1,P_2,P_3} \right)$ as the order parameter to describe the ferroelectric state in the PbTiO_3_ thin film. The temporal evolution of $P_i$ (*i* = 1–3) is calculated by minimizing the total free energy *F* with respect to $P_i$ via numerically solving the time-dependent Landau–Ginzburg–Devonshire (LGD) equations^37^,

1 $$\frac{{\partial P_i({\mathbf{x}},t)}}{{\partial t}} = - L\frac{{\delta F}}{{\delta P_i({\mathbf{x}},t)}},(i = 1\sim 3)$$

where **x** is the spatial position (with *x*-, *y*-, z- axes along the [100], [010], and [001] Cartesian coordinate directions), *t* is the time, *L* is the kinetic coefficient related to the domain wall mobility. The total free energy *F* of the PTO thin film includes the Landau, gradient, elastic, electrostatic, and flexoelectric energies, which is written as,

2 $${F = \mathop {\int}\limits_V {[\, f_{{\mathrm{lan}}}(P_i) + f_{{\mathrm{grad}}}(\nabla P_i) + f_{{\mathrm{elas}}}(P_i,\varepsilon _{ij}) + f_{{\mathrm{elec}}}(P_i,E_i) + f_{{\mathrm{flexo}}}(P_i,\varepsilon _{kl},\nabla P_i,\nabla \varepsilon _{kl})]} dV}$$

where *V* is the total volume of the system, $\varepsilon _{ij}$ and $E_i$ denote the components of strain and electric fields, $\nabla$ is the gradient operator. Detailed expressions of each free-energy density can be found in the Ref. ^47^. Equation (1) is numerically solved using a semi-implicit spectral method^52^ based on a 3D geometry sampled on a 128Δ*x* × 128Δ*y* × 32Δ*z* system size, with Δ*x* = Δ*y* = Δ*z* = 1.0 nm. The thickness of the film, substrate, and air are 20Δ*z*, 10Δ*z*, and 2Δ*z*, respectively. The temperature is *T* = 25 °C, and an isotropic relative dielectric constant (*κ*_ii_) is chosen to be 50. The gradient-energy coefficients are set to be $G_{11}/G_{110} = 0.6$, while $G_{110} = 1.73 \times 10^{ - 10}\,{\mathrm{C}}^{{\mathrm{ - 2}}}\,{\mathrm{m}}^{\mathrm{4}}\,{\mathrm{N}}$. The Landau coefficients, electrostrictive coefficients, and elastic-compliance constants are collected from Ref. ^53^. The flexoelectric energy can be written as^54^

3 $$f_{{\mathrm{flexo}}} = \frac{{F_{ijkl}}}{2}(\varepsilon _{ij}\frac{{\partial P_k}}{{\partial x_l}} - P_k\frac{{\partial \varepsilon _{ij}}}{{\partial x_l}})$$

where, $F_{ijkl}^{}(i = 1\sim 3)$ are the flexoelectric coupling coefficients. In our previous work we found that the longitudinal (*F*_11_), shear (*F*_12_) and transverse (*F*_44_) flexoelectric coefficients could affect the polarization tilt near the domain wall/substrate junctions, where *F*_ij_’s are the three independent flexoelectric coupling coefficients in the cubic system using Voigt notation, i.e., *F*_11_ = *F*_1111_, *F*_12_ = *F*_1122_, and *F*_44_ = 2*F*_1221_. Due to the uncertainties of the magnitude of *F*_ij_’s, we use *F*_11_ = 1.0 × 10^−11^ (Vm^2^ N^−1^) and assume *F*_12_ = *F*_44_ = 0 for simplicity based on literature^52^. We model the scanning-probe tip as a spherical indenter on the film surface, and define the normal stress distribution on top surface based on the Hertzian model,

4 $$\sigma _{33}^{{\mathrm{tip}}}(r) = \left\{ {\begin{array}{*{20}{c}} { - \frac{{3F}}{{2\pi a^2}}\sqrt {1 - \frac{{r^2}}{{a^2}}} } & {(r \le a)} \\ {0.0} & {(r \ge a)} \end{array}} \right.$$

in which *F* is the mechanical load, *a* is the radius of the tip-sample contact area, and $r = \sqrt {(x - x_0)^2 + (y - y_0)^2}$ is the distance from any points (*x,y*) inside the contact area to the tip center (*x*_0_,*y*_0_).

## Supplementary information

Supplementary Information

## Data Availability

The data that support the findings of this study are available from the corresponding author upon reasonable request.
