# Supplementary material for: Mechanical-force-induced non-local collective ferroelastic switching in epitaxial lead-titanate thin films
Source: Nat Commun. 2019 Sep 2;10:3951. doi: 10.1038/s41467-019-11825-2 (PMC6718682; doi:10.1038/s41467-019-11825-2)
Supplement: Supplementary file 1 — Supplementary Information [file 41467_2019_11825_MOESM1_ESM.pdf]

Supplementary Information

Supplementary Note 1. Theoretical calculations of domain coexistence

The Landau-Ginsburg-Devonshire-type phenomenological theory with the free energy expanded up to the sixth orders in polarization is employed to study the coexistence of different domain configurations in a thin film subjected to epitaxial strain. We assume that the misfit strain is epitaxially uniform and transversely isotropic. The renormalized thermodynamic potential after the Legendre transformation of the Gibbs free energy can be expressed with respect to the primary order parameters of polarization P_{i} and internal mechanical stresses σ_{i} in the film as[1]

$$\begin{aligned} F = {} & \alpha_1 (P_1^2 + P_2^2 + P_3^2) + \alpha_{11}(P_1^4 + P_2^4 + P_3^4) + \alpha_{12}(P_1^2 P_2^2 + P_2^2 P_3^2 + P_3^2 P_1^2) + \alpha_{123} P_1^2 P_2^2 P_3^2 \\ & + \alpha_{111}(P_1^6 + P_2^6 + P_3^6) + \alpha_{112}[P_1^2(P_2^4 + P_3^4) + P_2^2(P_3^4 + P_1^4) + P_3^2(P_1^4 + P_2^4)] \\ & + \tfrac{1}{2} s_{11}(\sigma_1^2 + \sigma_2^2 + \sigma_3^2) + s_{12}(\sigma_1\sigma_2 + \sigma_2\sigma_3 + \sigma_3\sigma_1) + \tfrac{1}{2} s_{44}(\sigma_4^2 + \sigma_5^2 + \sigma_6^2) \end{aligned} \qquad (1)$$

where α_1, α_{11}, α_{12}, α_{111}, α_{112}, and α_{123} are the linear and nonlinear dielectric stiffness coefficients, s_{ij} and σ_i are the elastic compliances and mechanical stresses, respectively. Since the mechanical stress σ_{i} is related to the elastic strains including the total strain and the phase transition strain, *F* also takes into account the coupling between the polarization and elastic strain through the total elastic strain. Parameters for our calculations are taken from Refs[1,2].

For pure *c* domains, the spontaneous polarizations can be calculated as[3]:

$$P_c^2 = \frac{-\alpha_{33}^*}{3\alpha_{111}} + \left(\frac{\alpha_{33}^{*2}}{9\alpha_{111}^2} - \frac{-\alpha_3^*}{3\alpha_{111}} \right)^{1/2} \qquad (2)$$

$$F_c = \frac{S_m^2}{s_{11} + s_{12}} + \alpha_3^* P_{ac}^2 + \alpha_{33}^* P_{ac}^4 + \alpha_{111} P_{ac}^6 \qquad (3)$$

with $\alpha_3^{**} = \alpha_1 - 2Q_{12}S_m / (s_{11} + s_{12})$ and $\alpha_{33}^{**} = \alpha_{11} + Q_{12}{}^2 / (s_{11} + s_{12})$.

The elastic interactions between adjacent domains should be considered to calculate the free energies for a_1/a_2 and *c/a* domain configurations. Following the polydomain theory[3], the spontaneous polarization and the corresponding free energies for *c/a* domains can be calculated by using the following expression:

$$P_{ca}^2 = \frac{-\alpha_{33}^{**}}{3\alpha_{111}} + \left(\frac{\alpha_{33}^{**2}}{9\alpha_{111}^2} - \frac{-\alpha_3^{**}}{3\alpha_{111}} \right)^{1/2} \tag{4}$$

$$F_{ac} = \frac{S_m^2}{2s_{11}} + \alpha_3^{**} P_{ac}^2 + \alpha_{33}^{**} P_{ac}^4 + \alpha_{111} P_{ac}^6 \tag{5}$$

with $\alpha_3^{**} = \alpha_1 - Q_{12} S_m / s_{11}$ and $\alpha_{33}^{**} = \alpha_{11} + Q_{12}{}^2 / 2s_{11}$.

Similarly, the analytical expression of the spontaneous polarization and the corresponding free energies for a_1/a_2 domains are:

$$P_{aa}^2 = \frac{-\alpha_{11}^{**}}{3\alpha_{111}} + \left(\frac{\alpha_{11}^{**2}}{9\alpha_{111}^2} - \frac{\alpha_1^{*}}{3\alpha_{111}} \right)^{1/2} \tag{6}$$

$$F_{ac} = \frac{S_m^2}{s_{11} + s_{12}} + \alpha_1^{*} P_{aa}^2 + \alpha_{11}^{**} P_{aa}^4 + \alpha_{111} P_{aa}^6 \tag{7}$$

with $\alpha_1^{*} = \alpha_1 - (Q_{11} + Q_{12}) S_m / (s_{11} + s_{12})$ and $\alpha_{11}^{**} = \alpha_{11} + (Q_{11} + Q_{12})^2 / [4(s_{11} + s_{12})]$.

To examine the possible existence of each stable or metastable states, Hessian's functions and canonical distribution within the framework of statistical mechanics are adopted to calculate the corresponding distribution probability. For each statistically equivalent ensembles *i*, the distribution probability of the ensemble being in the energy level G_i can be written as:

$$\gamma_i \propto \exp(-\frac{G_i - G_0}{kt}) \tag{8}$$

where *k* is the Boltzmann's constant, *t* is temperature. $G_i = F_i V_i$ is the energy for the ith low-temperature phase with F_i the system free energy densities and V_i the corresponding volume. G_0 is the energy for the ground state. The existing fractions for each phase are

$f_i = \gamma_i / (\gamma_c + \gamma_{aa} + \gamma_{ca})$ with $\sum f_i = 1$. (i=c, aa, and ca refers to the possible existing c domain, a_1/a_2, and c/a domain structures). Free energy densities and the corresponding existing fractions of each possible structure indicate that a_1/a_2 domains and c/a domains coexist with equal existing fraction near a tensile strain of 0.46% (Supplementary Figure 1). With further increase of the tensile strain, a_1/a_2 domains increases but still can coexist with c/a domains in a large range of strain condition.

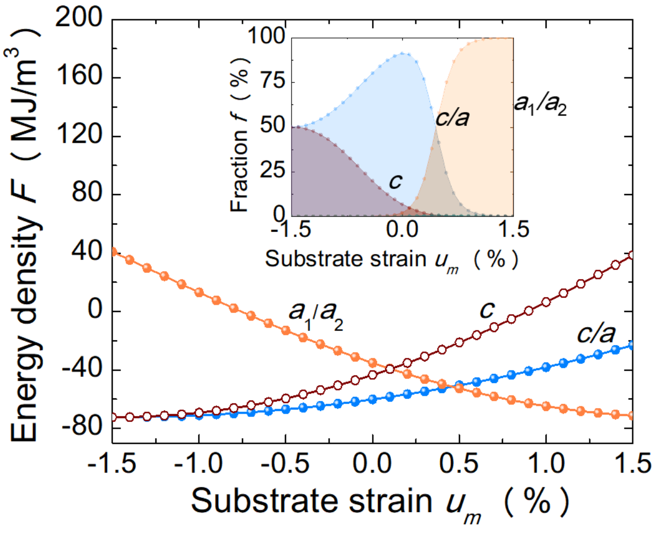

Supplementary Figure 1 | Strain evolution of polar structures. Energy densities of possible existing domain structures with respect to misfit strain. Insert is the existing fraction of c, a_1/a_2 and c/a domains.

Supplementary Note 2. Structural characterization using X-ray diffraction

Wide-angle θ-2θ X-ray diffraction pattern shows the epitaxial growth of the heterostructures (Supplementary Figure 2). Besides the diffraction peaks from $SmScO_3$ substrates, only 00*l* or *l*00 peaks from $PbTiO_3$ films are observed, suggesting epitaxial growth with the coexistence of c and a domains. Note that because the lattice of $(Ba_{0.5}Sr_{0.5})RuO_3$ electrode is very close to the $SmScO_3$ substrate, its diffraction peak overlaps with the substrate peak.

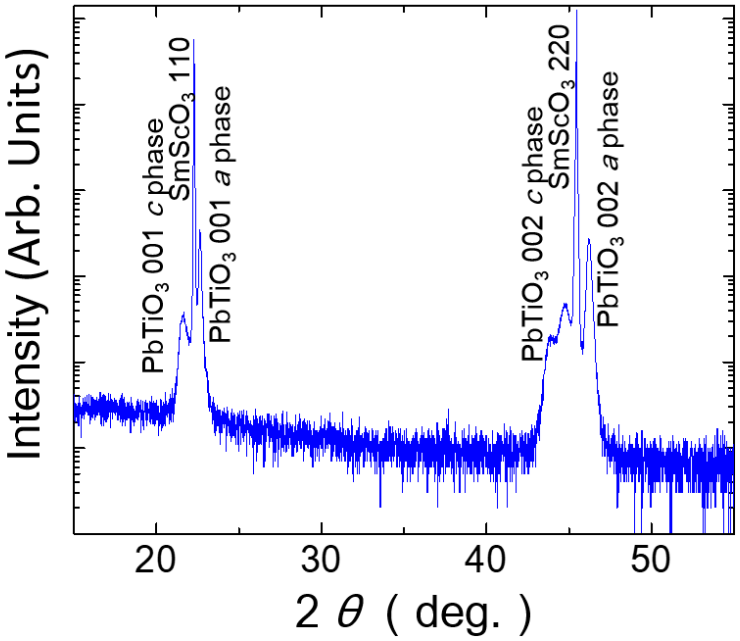

Supplementary Figure 2 | X-ray diffraction characterization. *θ*-2*θ* X-ray diffraction patterns for $PbTiO_3$ film deposited on $(Ba_{0.5}Sr_{0.5})RuO_3$-buffered $SmScO_3$ substrate.

Domain structures of the 70 nm thick $PbTiO_3$ films grown on $(Ba_{0.5}Sr_{0.5})RuO_3$-buffered $SmScO_3$ substrates is shown by XRD Reciprocal space mapping (RSM) studies about the on-axis 003-diffraction condition (Supplementary Figure 3). There is more than one diffraction peak from the $PbTiO_3$ film, consistent with the coexistence of *a* and *c* domains. Diffraction peaks from a_1/a_2 domains [L ~ 3.052 r.l.u. (reciprocal lattice unit), with out-of-plane lattice parameter of 3.922 Å] are found to have the same *H* value as that of the substrate, indicating that there is no tilt between the (001) plane of these domains and the substrate. Besides the untilted peaks from a_1/a_2 domains, two additional sets of diffraction peaks are observed which included two sets of peak pairs with the same *L* value but opposite *H* values: the first set with *L* ~2.906 r.l.u. corresponds to tilted *c* domains in *c*/*a* variants which has an out-of-plane lattice parameter of 4.120 Å, and it is tilted by an angle of ±1.8° (*H* ~ ±0.06 r.l.u.) into the [100] direction with respect to the substrate surface normal. The second set of two peaks with *L* ~

3.06 r.l.u. corresponds to the tilted *a* domains in *c*/*a* domains which has an out-of-plane lattice parameter of 3.912 Å and a tilt angle of ±1.7° (H ~ ±0.09 r.l.u.) along the [100] direction.

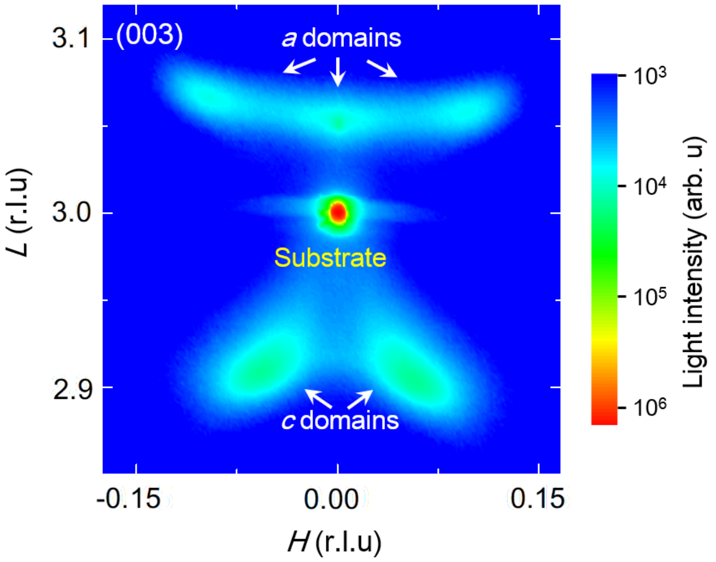

Supplementary Figure 3 | XRD Reciprocal space mapping (RSM) studies. On-axis RSM about the 003-diffraction condition of a $PbTiO_3/SrRuO_3/SmScO_3$ $(110)_O$ heterostructure.

Supplementary Note 3. Piezoelectric force microscopy studies of the as-grown films

The piezoelectric force microscopy studies are carried out on a MFP-3D (Asylum Research) with DART mode using Ir/Pt-coated conductive tips (Nanosensor, PPP-NCLPt). A coexistence of a_1/a_2 domain and *c*/*a* domains are confirmed in the out-of-plane (OOP) and in-plane (IP) PFM images (Supplementary Figure 4). The bright stripe region with high out-of-plane response are *c*/*a* domains (Supplementary Figure 4a) and most of the *c* domains are downward (c^-, Supplementary Figure 4b). Topography of *c*/*a* stripes are higher than that of the a_1/a_2 domains (insert in Supplementary Figure 4b). The dark region with low out-of-plane response (Supplementary Figure 4a) but high in-plane response (Supplementary Figure 4c) corresponds to in-plane a_1/a_2 domains (Supplementary Figures. 4c,d).

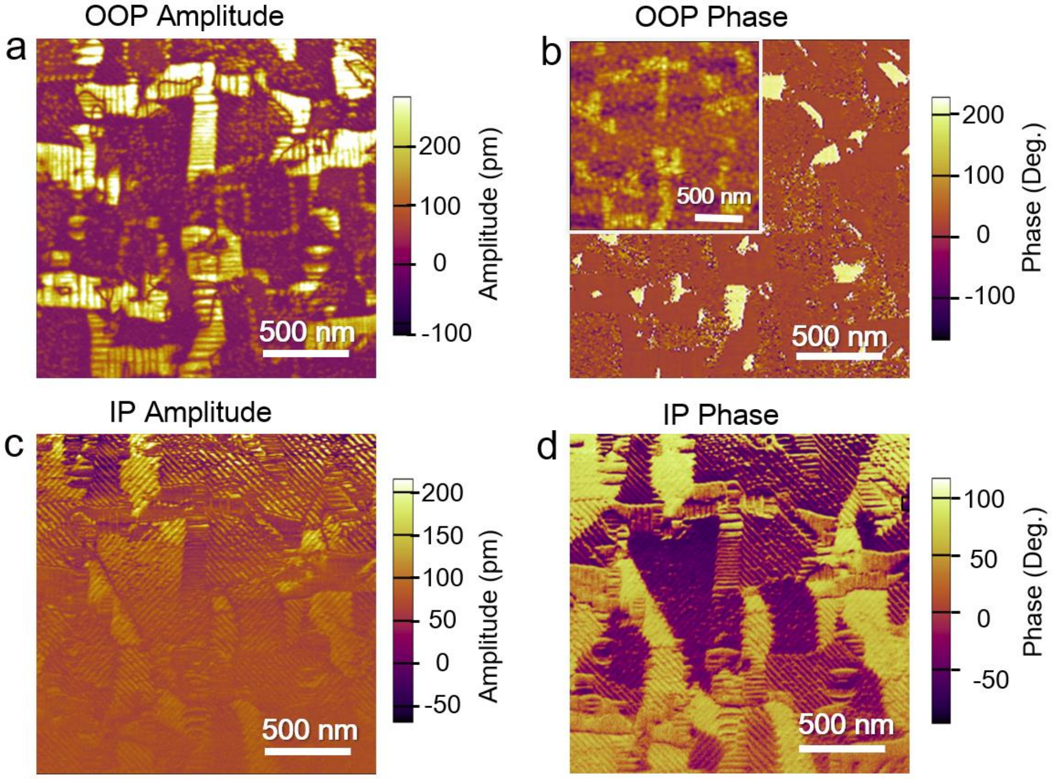

Supplementary Figure 4 | Piezoelectric force microscopy studies of the thin film. PFM scan of the $PbTiO_3/SrRuO_3/SmScO_3$ $(110)_O$ film with an area of 2 μm×2 μm. **a**, out-of-plane (OOP) PFM amplitude, **b**, OOP phase, insert in **b** is the topography, **c**, In-plane (IP) amplitude, **d**, IP phase. Scale bar is 500 nm.

Supplementary Note 4. Piezoelectric force microscopy studies of domain switching

To check the domain switching area induced by the four-point tip force located on the corner of 1 μm×1 μm, we scan a larger area with the same center of the force mapping area. As shown in a scanned area of 5 μm×5 μm, the height, out-of-plane (OPP) amplitude and phase images all clearly show that a fairly large area of about 2 μm×2 μm is affected by the tip force with driving voltage of 2 V (Supplementary Figure 5).

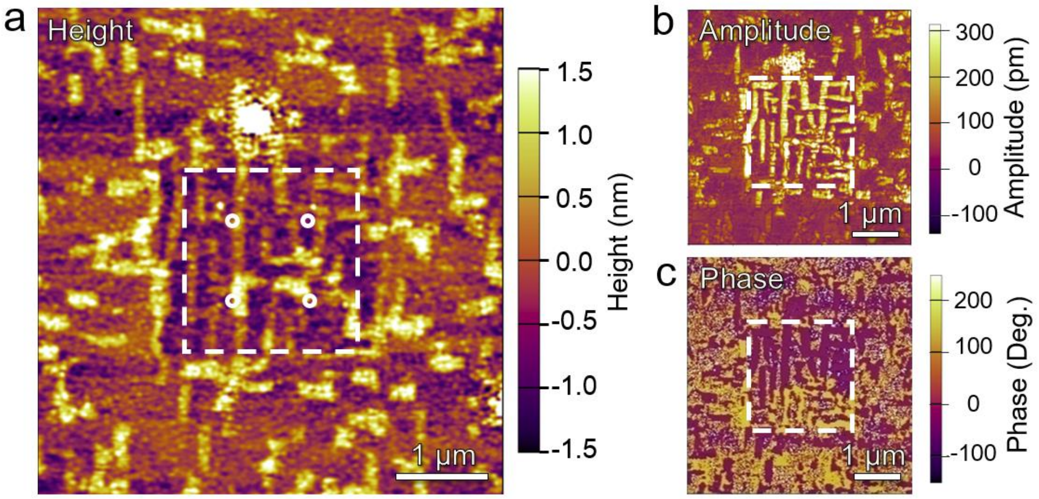

Supplementary Figure 5 | Domain switching propagation after the application of four-point local force. a, piezoelectric force microscopy (PFM) scan of height of 5 μm×5 μm area surrounding the tip force located on the four point as noted by the white circles. Responses arise in an area of about 2 μm×2 μm as noted by the white dished square. **b** and **c** are the corresponding amplitude and phase images, respectively.

Supplementary Note 5. Illustration of "collective ferroelastic switching"

In our experiment, the applied stress (force) is completed in, for example, a 2 × 2 array of points (the tip radius is ~25nm) at the corners of a 1 × 1 μm area within a 2 × 2 μm scanned area (Supplementary Figures 6a,b). A dramatic change in the domain structures, even well away (microns) from the tip contact area, has occurred after application of a stress (Supplementary Figure 6). That is, the change can extend across nearly the entire 2 × 2 μm scanned area (4×10^6 nm^2 area) when the force is applied only in a small fraction of that area (*e.g.*, the tip-sample contact area has a radius of ~110 nm under force of 600 nN on four points, that is just ~3.8% of the entire switched area; Supplementary Figure 6d). As a result, we consider it as and refer to it as a nonlocal response.

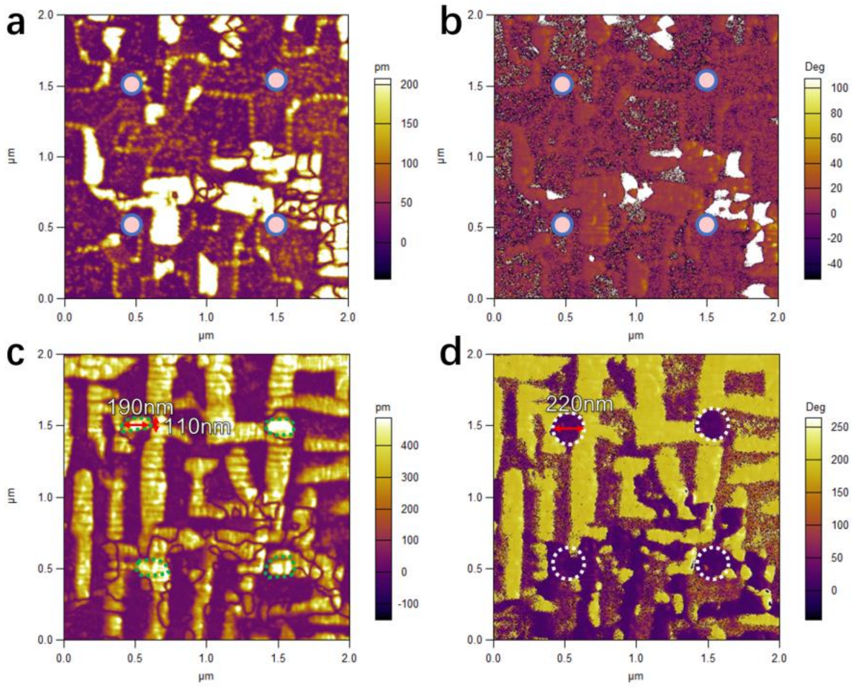

Supplementary Figure 6 | Mechanical-force-induced non-local, large-area ferroelastic switching. Amplitude and out-of-plane PFM images of the films **a**, and **b**, before the application of local four-point force as noted by four shaded circles. Amplitude and out-of-plane PFM images **c**, and **d**, after the four-point force mapping. The tip-sample contact areas and sizes are circled by the dotted line in the amplitude and out-of-plane PFM images **c**, and **d**, respectively.

To exclude the possible emergence of domains induced by the long-range stress field we further verify the stress state and the stressed area in the film under the scanning-probe tip using finite-element methods. To simplify our calculation, the elastic field is simulated by using isotropic materials constants. Here, the Young's module is 1.3×10^{11} Pa, and Poisson's ratio is 0.3. The size of the thin plate is set as 2 × 2 × 0.1 µm. The tip force is simulated by using a pressure applied on a circle area with a radius of 25 nm on the four corners of 1 × 1 µm on the film surface. We assume that the substrate is rigid enough that all the energy applied by the tip is absorbed by the film and transforms to elastic changes, thus the substrate on the boundary can be fixed with zero displacements. As shown in the commonly used von Mises stress contour map (Supplementary Figure 7a), and elastic stresses (Supplementary Figures 7b-d),

only local area surrounding the tip is affected by the tip force. Based on this simulation result, we conclude that it is not possible for such tip-induced stress field to propagate widely (across the entire area over which switching is observed) in a normal elastic material, and enable the domain mergence in a large distance. We should note that the value colored in blue is on the order of 10^3, and can be ignored as noise compared with the force on the order of 10^8.

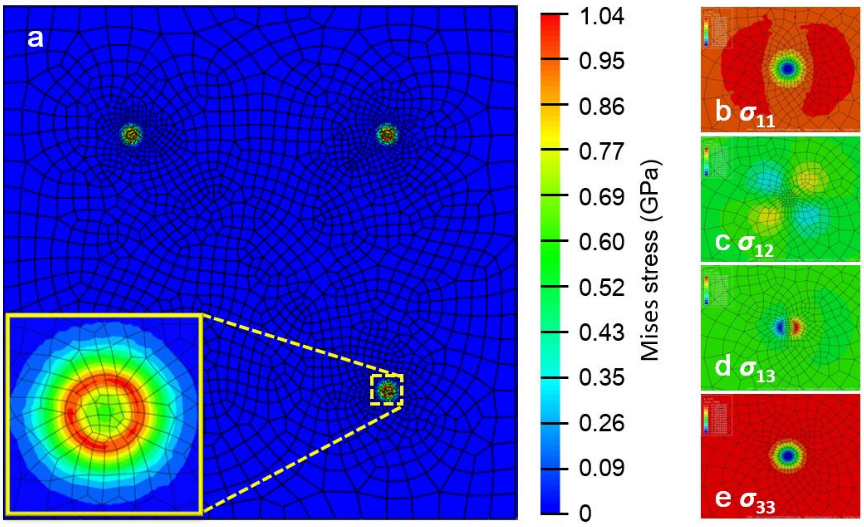

Supplementary Figure 7 | Stress distribution in film with tip force on four points on the surface. Pressure of 1.2 GPa comparable of 600 nN was applied on a circle area with radius of 25 nm at the corner of 1 × 1 μm on the surface. **a,** von Mises stress distribution. Insert is the amplification of the squared area. **b-d**, the corresponding amplification of stress components of σ_{11}, σ_{12}, σ_{13}, and σ_{33}.

We should note that this collective domain switching effect can exist in systems with degenerate domains structures which can be easily switched with small stimuli due to the small energy barriers between the states. Thus, the existence of this effect is likely in materials positioned (via strain, chemistry, etc.) near such structural/domain structure boundaries. Without this near energetic degeneracy between the phases, such effects are not expected. For example, $PbTiO_3$ thin films grown under the same conditions but on $SrTiO_3$ (001) substrates wherein the lattice mismatch is < 0.2% (with compressive misfit strain of ~-1%, and a strong preference for majority *c* and minority *a* domain structures) do not exhibit such nonlocal

domain switching effects (Supplementary Figure 8). We believe that there can be similar effects in other ferroelectric or ferroelastic systems in similar conditions.

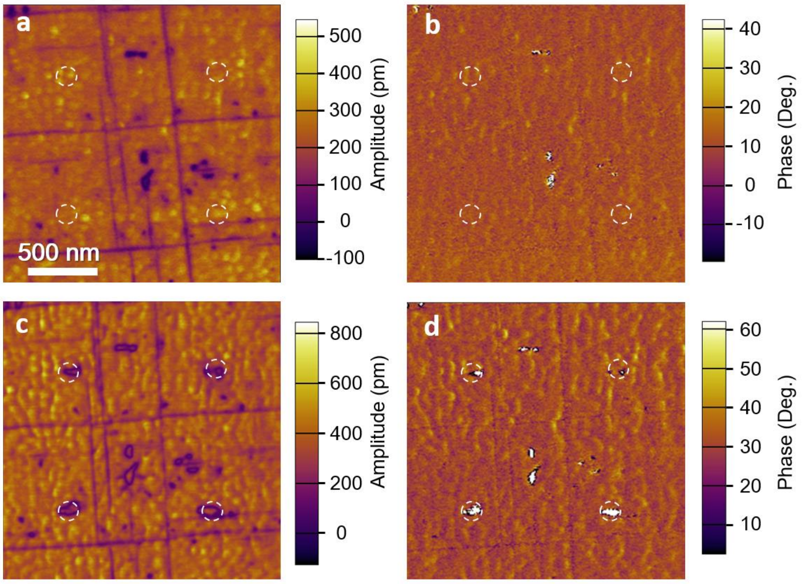

Supplementary Figure 8 | Application of tip force on $PbTiO_3$ thin films on $SrTiO_3$ substrate. **a**, **b,** and **c, d,** are PFM images of the out-of-plane amplitude, and phase before and after force with driving voltage of 2V, respectively.

Supplementary Note 6. Phase-field simulation of elastic, flexoelectric fields

Phase-field simulation of domain switching under mechanical tip pressures are carried out by taking polarization vector $P_i = (P_x, P_y, P_z)$ as order parameters. The temporal evolution of P_i is calculated by minimizing the total free energy with respect to P_i via numerically solving the time-dependent Landau–Ginzburg–Devonshire (LGD) equations. Using a semi-implicit spectral method, the polarization is calculated based on a 3D geometry sampled on a $128\Delta x \times 128\Delta y \times 32\Delta z$ system size, with $\Delta x = \Delta y = \Delta z = 1.0$ nm. The thickness of the film, substrate and air are $20\Delta z$, $10\Delta z$ and $2\Delta z$ respectively. We start from $PbTiO_3$ thin film consisting of alternating a_1/a_2 domain structures under 1.0% tensile strain in equilibrium state. The misfit strain is then reduced to +0.5% with the corresponding equilibrium states as shown

in Figure 4. Under a probe tip force with load of about 600 nN exerted concurrently at four different locations on the film surface (x_0, y_0) = (32, 32), (96, 32), (32, 96), (96, 96) (unit: nm), the final stable stress field can be calculated (Supplementary Figure 9).

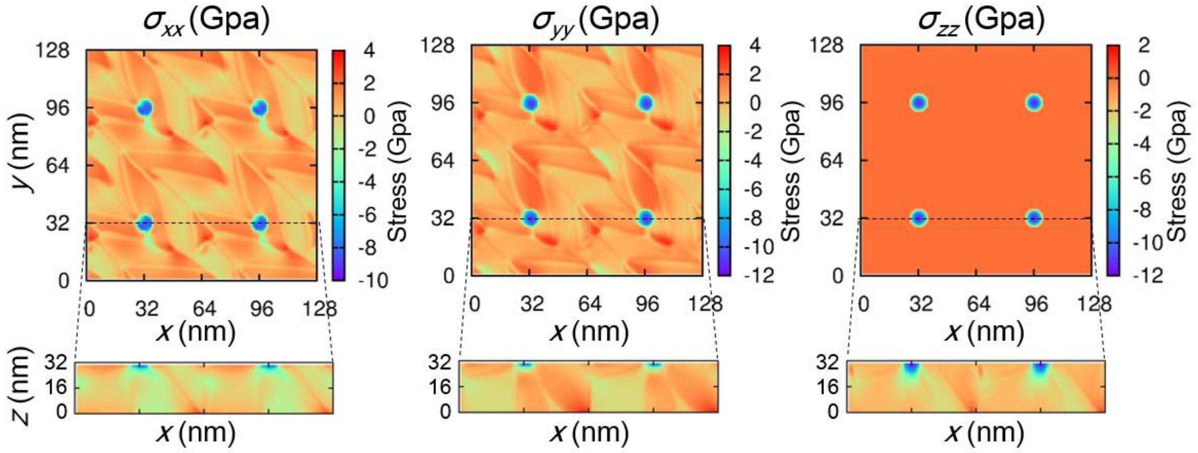

Supplementary Figure 9 | Phase-field simulation of stress field. Stress field distributions in films under tensile strain of 0.5% after the application of probe tip force of 600 nN.

We can further calculate the induced flexoelectric field in the film of the steady state as following:

$$
\begin{aligned}
E_1^f &= F_{11}\frac{\partial\sigma_1}{\partial x_1} + F_{12}\left(\frac{\partial\sigma_2}{\partial x_1} + \frac{\partial\sigma_3}{\partial x_1}\right) + F_{44}\left(\frac{\partial\sigma_5}{\partial x_3} + \frac{\partial\sigma_6}{\partial x_2}\right) \\
E_2^f &= F_{11}\frac{\partial\sigma_2}{\partial x_2} + F_{12}\left(\frac{\partial\sigma_3}{\partial x_2} + \frac{\partial\sigma_1}{\partial x_2}\right) + F_{44}\left(\frac{\partial\sigma_6}{\partial x_1} + \frac{\partial\sigma_4}{\partial x_3}\right) \\
E_3^f &= F_{11}\frac{\partial\sigma_3}{\partial x_3} + F_{12}\left(\frac{\partial\sigma_1}{\partial x_3} + \frac{\partial\sigma_2}{\partial x_3}\right) + F_{44}\left(\frac{\partial\sigma_4}{\partial x_2} + \frac{\partial\sigma_5}{\partial x_1}\right)
\end{aligned} \tag{9}
$$

where, F_{ij} $(i = 1 \sim 4)$ are the induced flexoelectric coefficient. Since the anisotropic terms are usually small, we only consider the first terms.

With $F_{11} = 1.0\times10^{-11}$ Vm^{2}N^{-1} and $F_{12} = F_{44} = 0$, we could be able to simulate the flexoelectric field distribution. For film under misfit strain of 0.5%, the induced flexoelectric field of the final stable states indicate that the film is under large strain gradient with the application of tip force, thus inducing a strong flexoelectric field beneath the tip area, which actually favors the

formation of *c* domain (Supplementary Figure 10). However, the role of the elastic compression is more evident than that of the flexoelectric field, thus, *a* domain structures are still preferred beneath the tip. We should note that the situation in the experiments is more complex since the tip is sharp and possible damage may be caused during the tip engage onto the thin film. Close to the tip location, the large strain gradient of out-of-plane strain induce strong electric field along the film normal direction, which favor the formation of *c* domains (Supplementary Figure 10c). Next to this area, there are large in-plane strain gradient, which induce a pair of positive and negative electric fields and favor the formation of *a* domains next to the *c* domains (Supplementary Figures 10a,b).

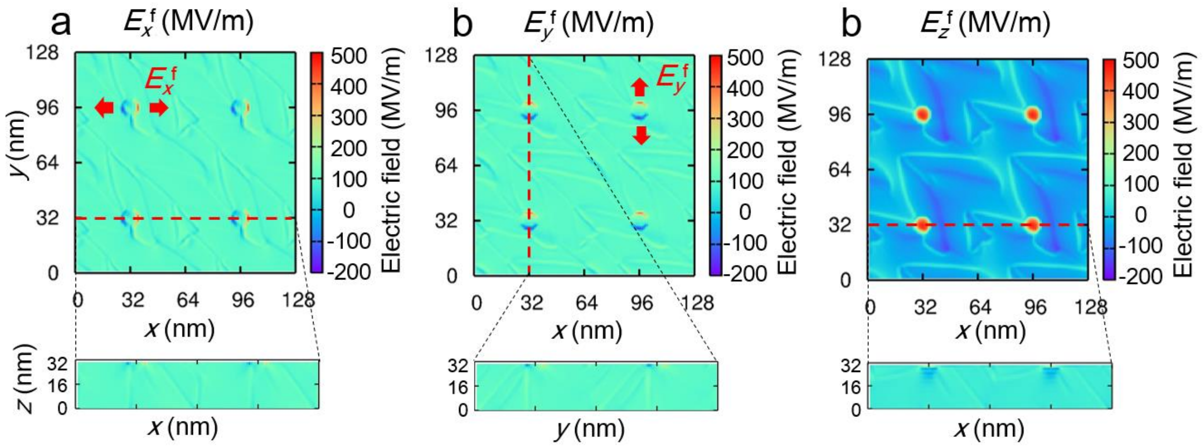

Supplementary Figure 10 | Phase-field simulation of flexoelectric-effect-induced electric field. Flexoelectric-effect-induced electric field distributions in films under tensile strain of 0.5% after the application of probe tip force of 600 nN. **a, b,** in-plane strain gradient induced a pair of positive and negative electric fields. **c,** large strain gradient of out-of-plane strain induced strong electric field along the film normal direction close to the tip location.

The Landau free energy, elastic energy, and electrostatic energy decrease the total energy during the formation of *c* domains, while the gradient energy increases the total energy at the domain boundaries with the increase of domain density (Supplementary Figure 11). Such collective domain switch are highly motivated by lowering the Landau free energy, elastic energy and electrostatic energy to maintain the lowest total energy.

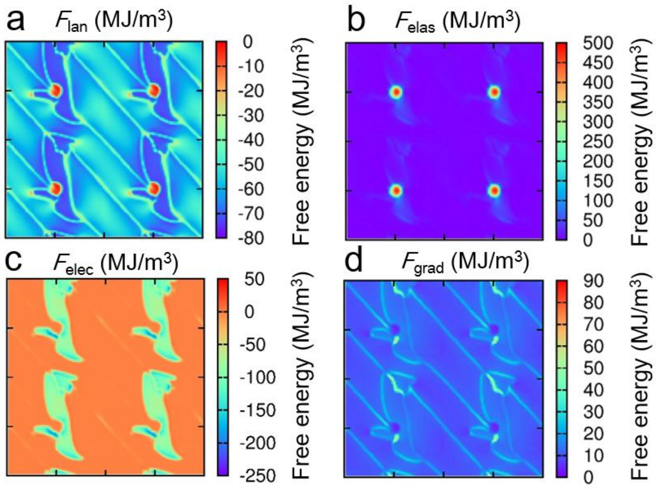

Supplementary Figure 11 | Phase-field simulation of the energy density profiles on the top *x-y* plane of the film. The total free energy including **a,** Landau free energy density F_{lan}, **b,** elastic energy density F_{elas}, **c,** electrostatic energy density F_{elec}, and **d,** gradient energy density F_{grad} in film with tensile strain of 0.5% after the applied tip force.

Most significantly, the elastic field change dramatically with the domain evolution. Upon application of the four-point 600 nN tip force in the 2 × 2 array, *c* domains form near the tip loaded area as described ahead. With the formation of *c* domains, the elastic field dramatically changes with enhanced tensile in-plane strain (Supplementary Figure 12a) and out-of-plane compressive strain (Supplementary Figure 12b) surrounded the formed *c* domains, which favors the formation of *a* domains. Since the as-grown film is under high energy level with large tensile in-plane strain, and the formed *c/a* domains are energetically favorable, thus, nonlocal and collective domain switch from a_1/a_2 domain structure to *c/a* domain structure can be enabled and spread in a large area.

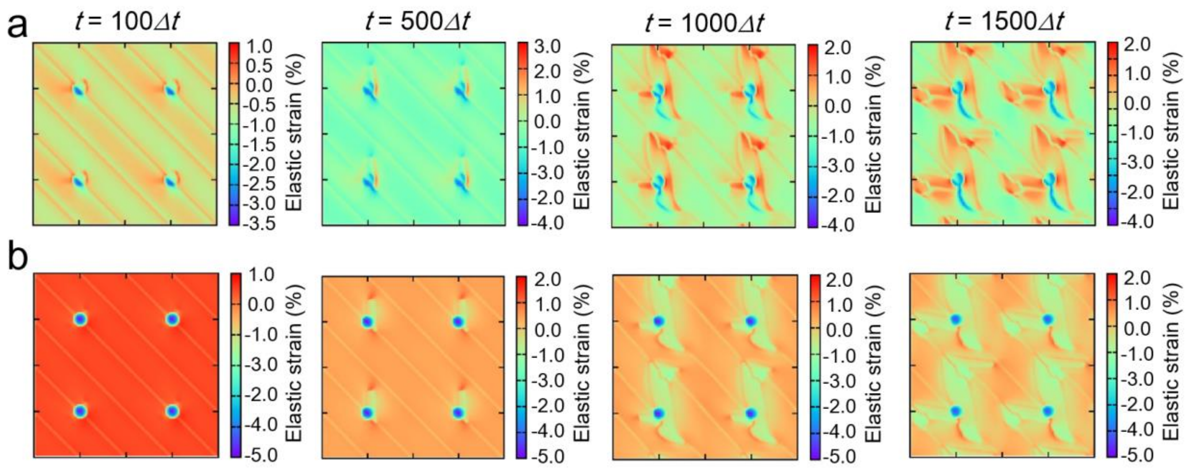

Supplementary Figure 12 | Phase-field simulation of the elastic strains during the domain evolution. Elastic strain evolution of **a,** in-plane strain, and **b,** out-of-plane strain in the film with tensile strain of 0.5% after the applied tip induced mechanical force.

Supplementary Note 7. Energy barrier from Landau phenomenological theory

The Landau free energy densities of the a_1/a_2 and *c/a* domain structures at high temperature of 500K and 300K are shown in (Supplementary Figure 13). We note that the Curie temperature for the films under strain of 0.46% is ~891 K (618℃). At high temperature, the a_1/a_2 domain structure dominates, and is kept during the cooling by the misfit strain until external perturbation is given. Since a_1/a_2 and *c/a* domains have equal free energy at 300K, *c/a* domains are favorable even without other external field, thus the a_1/a_2 domains under applied stress with a much higher energy potential can be abruptly switched into *c/a* domains with a collective, non-local behavior.

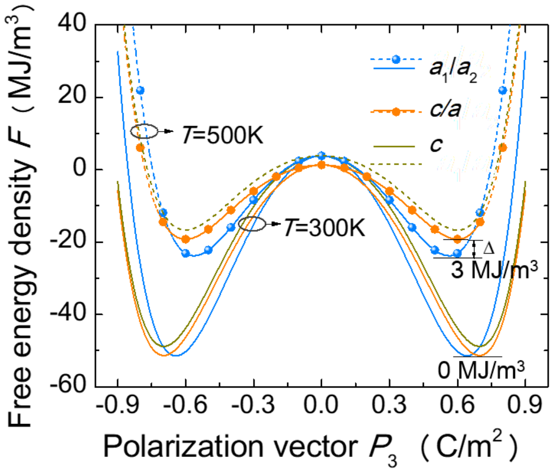

Supplementary Figure 13 | Energy barrier based on the Landau phenomenological theory. Landau free energy densities and phase structure transition barrier at high temperature and room temperature.

Supplementary Note 8. Phase-field simulation of domain structure under different misfit strains

In order to investigate the domain evolutions in other strain condition, the phase-field simulation of the equilibrium domain structures in the as-grown film before tip pressure is studied for reference (Supplementary Figure 14). Under compression or small tensile misfit strain, *c* domains dominate with isolated *a* domains in the *c* domain matrix (Supplementary Figure 14a). With the increase of substrate tensile strain, *a* domain increase and dense *c/a* domain structures can be observed at misfit strain of 0.1% (Supplementary Figure 14b). With further increase of the misfit strain, small part of a_1/a_2 domains emerge and coexist with *c/a* domains (Supplementary Figure 14c). So the a_1/a_2 domain is actually unstable even before the tip pressure when the misfit strain is below 0.2%. However, for film under misfit strain above 0.3%, a_1/a_2 domain is under quasi-steady state (Supplementary Figures 14d-f). With the further increase of tensile strain, the a_1/a_2 domains are more favorable until to be the most stable structure.

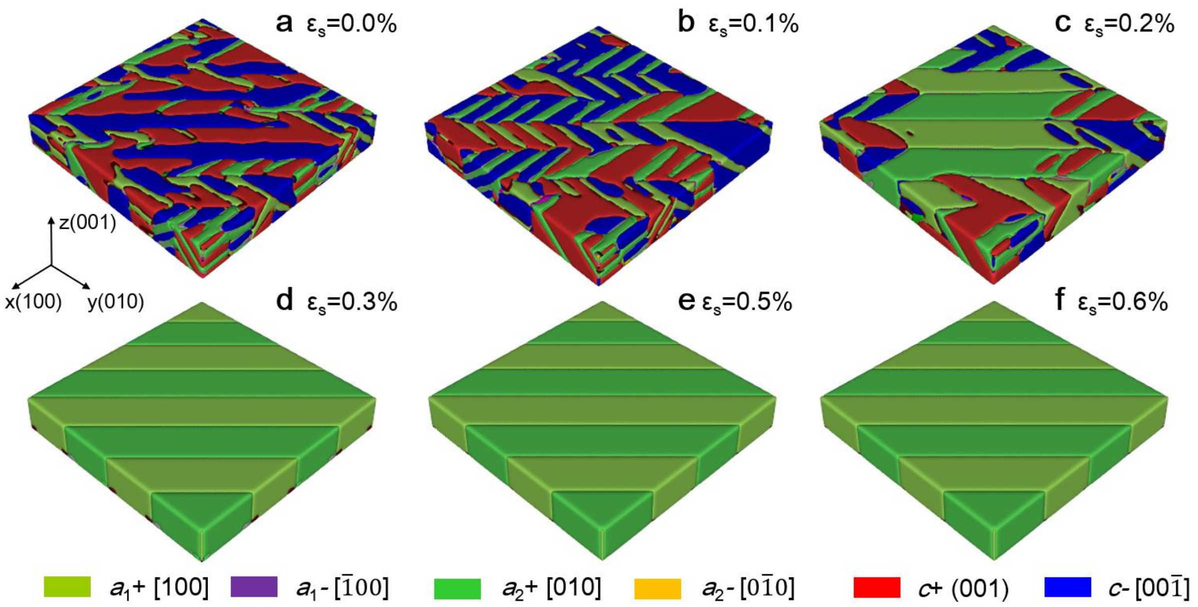

Supplementary Figure 14 | Phase-field simulation of equilibrium domain structures. Equilibrium domain structures in the as-grown film under various misfit strains before the tip pressure. **a,** under misfit strain of 0%, *c* domains dominate with isolated *a* domains in the *c* domain matrix. **b,** under misfit strain of 0.1%, *a* domain increase and dense *c/a* domain structures. **c,** under misfit strain of 0.2%, small part of a_1/a_2 domains emerge and coexist with *c/a* domains. **d-f,** under misfit strain of 0.3%-0.6%, a_1/a_2 domain is under quasi-steady state.

Supplementary Note 9. Thickness effects - phase-field simulations of domain structures

In the current phase-field simulation, we chose a simulation size of $128\Delta x \times 128\Delta x \times 32\Delta x$ (Δx = 1 nm) with periodic boundary conditions to simulate the non-local domain switching and compare with experiments. We believe this is a balance between computation accuracy and efficiency. Admittedly, our simulated domain size and film thickness are smaller than the actual samples. In order to explore if there was a thickness effect, we performed additional phase-field simulations for 20 nm, 30 nm, 50 nm, and 70 nm thick $PbTiO_3$ thin films under 0.5% and 1.0% tensile strains (Supplementary Figure 15). Despite different thickness of the film, the results show a similar trend. Under misfit strain of 0.5%, large area domain switching is observable; while under misfit strain of 1.0%, the initial domain structure is stable. It is also seen that the depth of the switched *c*+ and *c*- domains is around 30 nm. Therefore, we believe

our current simulations capture well the non-local domain switching dynamics as observed in experiment.

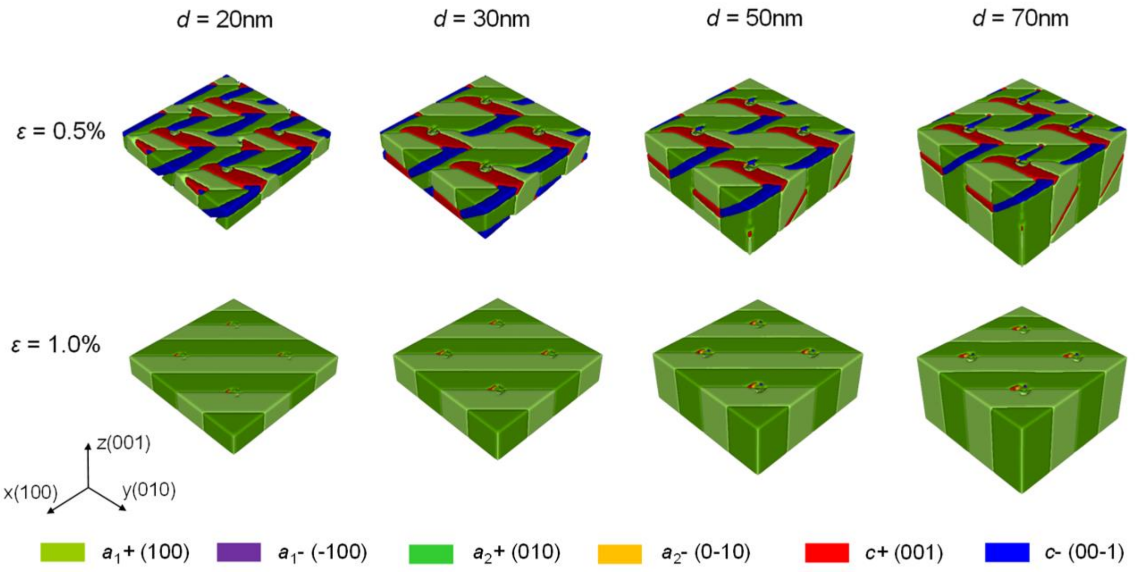

Supplementary Figure 15 | Phase field simulation of mechanical switching in $PbTiO_3$ thin film of different thickness and under different misfit strains. Equilibrium domain structures with different film thickness show a similar trend. Under misfit strain of 0.5%, large area domain switching is observed; while under misfit strain of 1.0%, the initial a_1/a_2 domain structure remain stable for all films.

Supplementary References

1 Pertsev, N. A. *et al*. Phase diagrams and physical properties of single-domain epitaxial $Pb(Zr_{1-x}Ti_x)O_3$ thin films. *Phys. Rev. B* **67**, 480-485 (2003).

2 Li, Y. L. *et al*. Ferroelectric domain morphologies of (001) $Pb(Zr_{1-x}Ti_x)O_3$ epitaxial thin films. *J. Appl. Phys.* **97**, 034112 (2005).

3 Kukhar, V. *et al*. Polarization states of polydomain epitaxial $Pb(Zr_{1-x}Ti_x)O_3$ thin films and their dielectric properties. *Phys. Rev. B* **73**, 214103 (2006).
